# Subunit-Specific Reactivity of Autoantibodies Against Laminin-332 Reveals Direct Inflammatory Mechanisms on Keratinocytes

**DOI:** 10.3389/fimmu.2021.775412

**Published:** 2021-11-25

**Authors:** Lei Bao, Jing Li, Farzan Solimani, Dario Didona, Payal M. Patel, Xiaoguang Li, Hua Qian, Norito Ishii, Takashi Hashimoto, Michael Hertl, Kyle T. Amber

**Affiliations:** ^1^ Department of Dermatology, University of Illinois at Chicago, Chicago, IL, United States; ^2^ Division of Dermatology, Rush University Medical Center, Chicago, IL, United States; ^3^ Department of Dermatology and Allergology, Philipps University, Marburg, Germany; ^4^ Department of Dermatology, Venereology and Allergology, Charitè–Universitätsmedizin Berlin, Corporate Member of Freie Universität Berlin, Humboldt-Universität zu Berlin, and Berlin Institute of Health, Berlin, Germany; ^5^ Central Laboratory, Dermatology Hospital of Jiangxi Province, Dermatology Institute of Jiangxi Province, and the Affiliated Dermatology Hospital of Nanchang University, Nanchang, China; ^6^ Department of Dermatology, Kurume University School of Medicine, and Kurume University Institute of Cutaneous Cell Biology, Kurume, Japan; ^7^ Department of Dermatology, Osaka City University Graduate School of Medicine, Osaka, Japan; ^8^ Department of Internal Medicine, Rush University Medical Center, Chicago, IL, United States

**Keywords:** laminin-332 pemphigoid, keratinocyte, RNA-seq, autoimmune blistering diseases, immunobullous disease

## Abstract

Laminin-332 pemphigoid is a rare and severe autoimmune blistering disease, caused by IgG autoantibodies targeting laminin-332 in the dermal-epidermal basement zone. Laminin-332 pemphigoid is characterized by variable inflammatory infiltrate and the predominance of non-complement-fixing antibodies. Given these findings, we hypothesized that IgG autoantibodies to laminin-332 directly resulted in keratinocyte expression of inflammatory factors. We performed RNA-seq on primary human keratinocytes treated with IgG from patients with laminin-332 pemphigoid. Genes for numerous cytokines and chemokines were upregulated, including CSF2, CSF3, CXCL1, CXCL5, CXCL3, CXCL8, CXCL10, CXCL1, IL6, IL7, IL15, IL23, IL32, IL37, TGFB2 as well as metalloproteases. Considering the pro-inflammatory and proteolytic effect of autoantibodies from patients with laminin-332 pemphigoid identified in our initial experiment, we next questioned whether the reactivity against specific laminin subunits dictates the inflammatory and proteolytic keratinocyte response. Then, we treated keratinocytes with IgG from a separate cohort of patients with reactivity against individual subunits of laminin-332. We identified upregulation of IL-1α, IL-6, IL-8, CXCL1, MMP9, TSLP, and GM-CSF at the protein level, most notably in keratinocytes treated with IgG from laminin β3-reactive patients. We for the first time demonstrated a pro-inflammatory response, similar to that described in keratinocytes treated with IgG autoantibodies from patients with bullous pemphigoid, providing novel insight into the pathogenesis of laminin-332 pemphigoid and laminin-332 biology.

## Introduction

Laminin-332 pemphigoid, a subtype of mucous membrane pemphigoid (MMP), is a rare and severe autoimmune blistering disease, caused by IgG autoantibodies targeting laminin-332 in the dermal-epidermal basement zone (BMZ) (1–3). Laminin-332 is an extracellular glycoprotein composed of the α3, β3, and γ2 subunits with a large G domain at the base containing epidermal growth factor-based repeats (4, 5). The α3 subunit is the most frequently targeted subunit in laminin-332 pemphigoid, followed by γ2 and β3 (6, 7). Laminin-332 pemphigoid has been associated with a more aggressive phenotype and with extensive laryngopharyngeal involvement (6, 8–11).

Several animal models have supported the pathogenicity of anti-laminin-332 IgG (12–14). The role of complement in inducing local inflammatory response and subsequent blistering remains unclear. For example, passive transfer experiments in neonatal mice demonstrate anti-laminin-332 IgG induced blistering, but a lack of local inflammation and significant mucosal involvement. In an adult mouse model of laminin-332 using anti-α3 antibodies, a more characteristic clinical phenotype with a local inflammatory response was appreciated in an Fc-dependent manner (14). Histologically, laminin-332 pemphigoid is characterized by variable inflammatory infiltrate (14, 15). It has also been reported that non-complement-fixing IgG antibodies against laminin 332 are the predominant class of autoantibodies deposited at the cutaneous BMZ in patients with laminin-332 pemphigoid (16). As such, it is likely that complement-independent inflammatory pathways exist in human disease.

Aside from their structural role, hemidesmosomal proteins may additionally regulate the local inflammatory response. For example, IgG autoantibodies to collagen XVII in bullous pemphigoid (BP) lead to internalization of collagen XVII in keratinocytes (17), with upregulation of the pro-inflammatory cytokines, IL-6 and IL-8. Genetic modification of collagen XVII additionally induces eosinophilia and keratinocyte expression of thymic stromal lymphopoietin (TSLP) (18). Notably, the presence of complement deposition in BP was inversely related to the presence of lesional neutrophils, thus suggesting a complement-independent inflammatory mechanism. As such, it is evident that numerous complement-independent pathways exist in BP (19).

Given the heterogeneity of cutaneous inflammation and the predominance of non-complement-fixing antibodies in laminin-332 pemphigoid, we hypothesized that IgG autoantibodies to laminin-332 directly resulted in keratinocyte expression of inflammatory factors.

## Materials and Methods

### Patients

All analyses of human materials were performed in accordance with the principles in the Declaration of Helsinki and approved by the ethical committee at the University of Illinois at Chicago, Rush University Medical Center, Philipps University, and Kurume University School of Medicine. Diagnosis of laminin-332 mucous membrane pemphigoid in the first cohort of patients was based on clinical presentation of MMP, direct immunofluorescence (DIF) demonstrating linear IgG deposition at the cutaneous BMZ, and with indirect immunofluorescence (IIF) against the dermal side of salt-split skin. All patients were screened and negative for BP180/BP230 antibodies (20). ELISA was performed using affinity purified native human laminin-332 (containing α3, β3, and γ2 subunits) from the squamous cell carcinoma cell line SCC25 as previously described (20). Diagnosis in the second cohort of patients was also based on DIF and salt-split IIF and/or immunoblots against each individual subunit of laminin-332. All sera were screened for the presence of BP180, BP230, and collagen 7 antibodies by ELISA, and p200 by immunoblotting. As epitope spreading is a common phenomenon, sera containing antibodies against BP180 or BP230 were not excluded from analysis as IIF showed only a dermal pattern. One patient additionally demonstrated autoantibodies to the p200 antigen, but this was considered to be epitope spreading, as the patient had aggressive mucous membrane disease, clinically most consistent with laminin-332 pemphigoid. Autoantibody profiles are shown in **Table 1**. Control human serum (Thermo Fisher Scientific, Waltham, MA, USA) was purchased.

**Table 1:** Laminin-332 pemphigoid patients with IgG reactivity against a single subunit of laminin-332.

ID	Age	Sex	IIF Dermal	Laminin Domain reactivity	Other autoantibodies
LAMA3-IgG1	85	M	+40	α3	–
LAMA3-IgG2	61	M	+10	α3	–
LAMA3-IgG3	63	M	+10	α3	BP180/BP230
LAMA3-IgG4	50	F	+10	α3	–
LAMB3-IgG1	NR	NR	+10	β3	BP180
LAMB3-IgG2	NR	NR	+10	β3	–
LAMB3-IgG3	49	F	+10	β3	–
LAMB3-IgG4	78	M	+10	β3	–
LAMC2-IgG1	56	F	–	γ2	–
LAMC2-IgG2	74	M	+10	γ2	–
LAMC2-IgG3	84	M	+10	γ2	P200
LAMC2-IgG4	69	M	+10	γ2	–

IIF, indirect immunofluorescence; NR, No record. IIF was performed on salt split human skin with serial dilution to 1:10 and 1:40. Laminin subunit reactivity was determined by immunoblotting. The presence of BP180, BP230, and collagen 7 antibodies was assessed by ELISA. Notably, neither patient with BP180 antibodies demonstrated epidermal staining. Immunoblotting was used to screen sera for reactivity against p200.

### IgG Purification

IgG extraction was performed using Pearl IgG Purification Kits (G-Biosciences, St. Louis, MO, USA) according to manufacturer’s instructions. Isolated IgG was subsequently washed and concentrated using Amicon Ultra-15 centrifugal filter units with a 100kDa filter in PBS (Millipore Sigma, Burlington, MA, USA).

### Cell Culture

Primary adult human keratinocytes (PHKs) (Thermo Fisher Scientific, Waltham, MA, USA) were cultured in EpiLife Medium with Human Keratinocyte Growth Supplement (Thermo Fisher Scientific, Waltham, MA, USA) in a humidified atmosphere of 5% CO^2^ at 37°. Fresh culture media were replaced every 48 h. When confluency reached 80-90%, cells were treated with the patients’ IgG (3.5 ug/ul) or normal human IgG for 24 h. Each sample represents a distinct culture. Finally, cells were washed twice with PBS and were stored at −80°C until RNA extraction. Culture supernatants were stored at −80°C until Luminex and ELISA assays were performed.

### RNA-Purification and RT-qPCR

Total RNA from PHKs was isolated using miRNeasy Mini Kit following the manufacturer’s instructions (Qiagen Inc., Germantown, MD, USA). On-Column DNase I Digestion was performed to prevent genomic DNA contamination. The RT and real-time PCR were performed as previously described (21). Briefly 1 μg of total RNA was reverse transcribed to 100 μl of cDNA. Samples were run in duplicates, and the PCR were carried out using a Stratagene Mx3000 real-time PCR machine (Agilent Technologies, Inc., Santa Clara, CA, USA). GAPDH was used as the internal reference. The standard –ddCT method was used to measure the relative mRNA expression. 2^-ΔΔCT^ values were compared using student’s T-test with significance defined as P < 0.05. Primers used in this study were synthesized by Integrated DNA Technologies Inc. (Coralville, IA, USA). Primers used are provided in **Supplementary Table 1**.

### Next Generation Sequencing Library Preparation and Sequencing

Library preparation was performed as previously described (22). RNA quantity and quality were assessed using a Nanodrop 1000 spectrophotometer (Nanodrop, Thermo Fisher Scientific, Waltham, MA, USA), with RNA integrity number and concentration checked using an Agilent 2100 Bioanalyzer (Agilent Technologies, Inc., Santa Clara, CA, USA). Isolated RNA samples were subsequently prepared for construction of transcriptome libraries. Poly (A) mRNA was isolated and enriched from total RNA using oligo (dT)-attached magnetic beads according to the manufacturer’s (Illumina, San Diego, CA, USA) instructions. Enriched and purified mRNA was subsequently broken into approximately 200 nt short mRNA with random hexamers used as primers to synthesize first-strand cDNA. Second-strand cDNA was synthesized in a buffer containing dNTPs, DNA polymerase I, and RNaseH. Suitable fragments were isolated and enriched by PCR amplification. Finally, the constructed cDNA libraries of the samples were sequenced using an Illumina HiSeq sequencing platform (San Diego, CA, USA). Raw data was uploaded to the Gene Expression Omnibus under the accession number GSE182644.

### Data Processing and Visualization

Filtered clear reads were mapped to the reference genome by HISAT2 (23). Mapped reads were converted to Fragments Per Kilobase of transcript per Million mapped reads (FPKM), and subsequently quantified with Cuffquant and Cuffnorm (24). DESeq was used for normalization and to analyze differentially expressed genes (25). Fold change ≥ 2 and false discovery rate <0.05 were set as screening criteria. Gene Ontology was assessed using the topGO package (26), utilizing the gene ontology database (http://geneontology.org/). Heat maps were generated using FPKM values using the Morpheus online server (https://software.broadinstitute.org/morpheus).

### Luminex and ELISA Assays

Luminex multiplex kits for IL-1a, IL-6, IL-8, CXCL1, matrix metalloproteinase 9 (MMP9), and TSLP were purchased from R&D systems (Minneapolis, MN, USA). Supernatants were diluted per manufacturer recommendations using a Flexmap 3D system (Luminex, Austin, TX, USA), performed at the Rush University Medical Center Biomarker Development Core. GM-CSF and HSPA5 ELISA were purchased from Thermo Fisher Scientific (Waltham, MA, USA) and MyBioSource (San Diego, CA, USA), respectively. Samples in Luminex and ELISA assays were run in duplicate. ELISAs were performed per the manufacturer’s instructions and read on a Tecan Spark plate imager (Tecan Group Ltd., Männedorf, Switzerland). Standard curves were calculated for all markers. To compare concentrations between controls and each laminin-subunit reactive, we used a one-way ANOVA with Tukey’s test (GraphPad Prism 6). Significance was set at P < 0.05. ClustVis was used to perform principal component analysis (27). Expression heat map was generated by normalizing protein concentrations and subsequently visualized using the heatmapper server (28).

## Results

### IgG From Patients With Laminin-332 Pemphigoid Results in Upregulation of Numerous Pro-Inflammatory Genes as Identified by RNA-Seq

**Figure 1:**
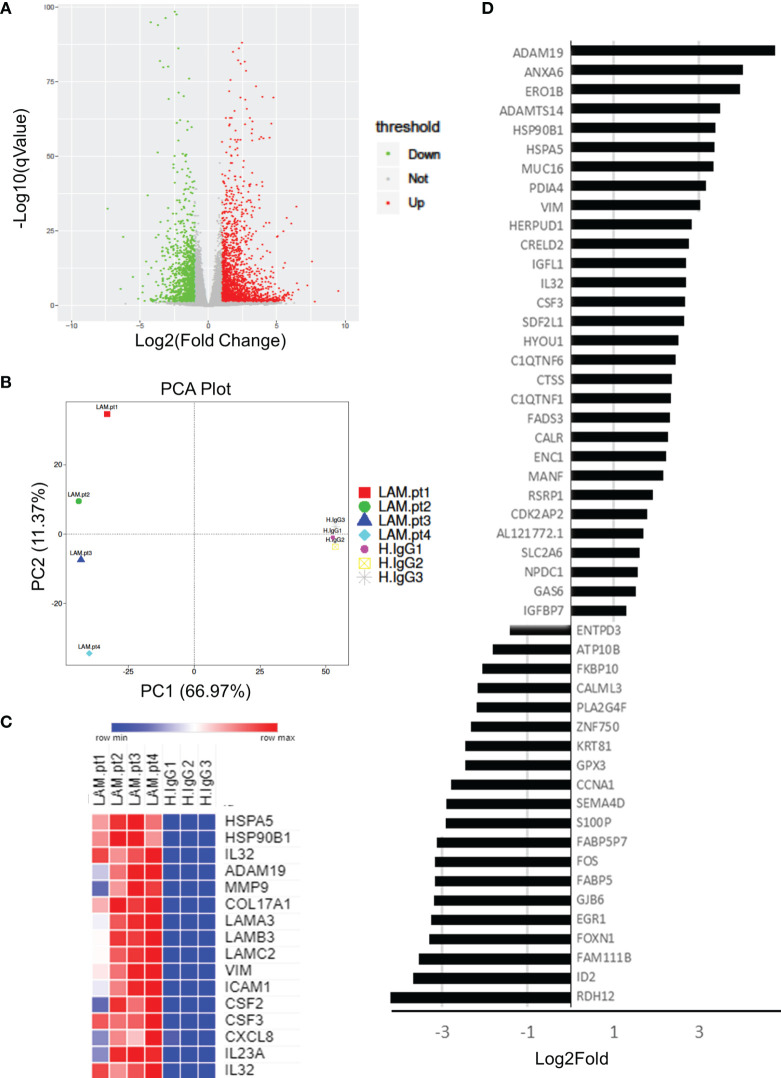
Primary human keratinocytes were treated for 24 hours with 3.5μg/μL of IgG from patients with laminin-332 pemphigoid (n=4), or control IgG. 3 different cultures were used for control IgG treatments. RNA-seq was performed on keratinocytes, resulting in **(A)** 3,228 genes to be differentially expressed (2,050 up, 1,179 down) as shown on a Volcano plot. **(B)** Principal component analysis demonstrating discrete clusters between laminin-332 IgG autoantibody treated keratinocytes (Lam.pt) as compared to health control (H.IgG). **(C)** Expression heat map of selected cytokines, chemokines, heat shock proteins, proteases, and structural proteins. **(D)** Top 50 differentially expressed genes as sorted by false discovery rate, shown from most highly upregulated down regulated.

Whole transcriptome sequencing was used to profile expression of primary keratinocytes treated with IgG from patients with untyped laminin-332 pemphigoid (n=4) or control serum (n=3). We obtained an average of 40.1 million clean reads per sample. An average of 96% genes mapped to the human genome. Principal component analysis demonstrates two distinct clusters between keratinocytes treated with laminin-332 IgG or control IgG (**Figure 1**). 3,228 genes are differentially expressed (2,050 up, 1,179 down). The top 50 most significantly differentially expressed genes by false discovery rate are shown in **(** **Figure 1** **)**. **Supplementary Table 2** summarizes all significantly differentially expressed genes.

As hypothesized, genes for numerous cytokines and chemokines were significantly upregulated, including CSF2, CSF3, CXCL1, CXCL5, CXCL3, CXCL8, CXCL10, CXCL1, IL6, IL7, IL15, IL23, IL32, IL37, TGFB2. Only TGFB3 and IL34 genes were downregulated. Interestingly, numerous heat shock genes were differentially expressed with HSP90B1, HSP90B2P, HSP90B3P, and HSPA5 highly upregulated, while HSPA2, HSPA4L, HSPA14, HSPB8, HSPE1P2, and HSPA6 were down regulated. Numerous proteases were additionally upregulated including MMP1, MMP2, MMP9, MMP10, MMP12, MMP14, MMP15, MMP19, MMP24, ADAM8, ADAM12, ADAM19, and ADAM21. Of these, MMP9 and ADAM19 were most highly upregulated. Lastly, hemidesmosomal genes COL17A1, COL7A1, LAMA3, LAMB3, and LAMC2 were upregulated. Heat map of select proteases, heat-shock proteins, cytokines, chemokines, and structural proteins are shown in (**Figure 1**).

### qPCR Validation of Select Genes Identified by Next Generation Sequencing

**Figure 2:**
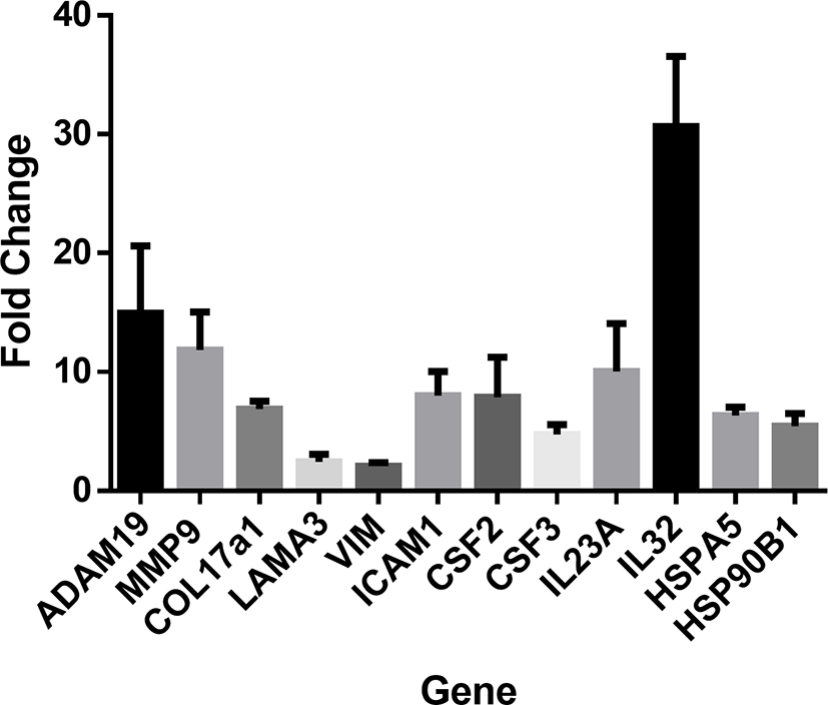
RT-PCR was performed to confirm next generation sequencing findings in several gene classes including cytokines, chemokines, heat shock proteins, proteases, and structural proteins. RT-PCR was performed in duplicates of laminin (n=4) or control cultures (n=3). Upregulation of ADAM19, MMP9, COL17A1, LAMA3, VIM, ICAM1, CSF2, CSF3, IL23A, IL32, HSPA5, and HSP90B1. Values indicate fold-change of laminin treated keratinocytes (n=4) relative to control treated cultures (n=3). Student’s T-test was used. All genes were confirmed as significant with P < 0.05.

To confirm next generation sequencing findings, RT-qPCR was performed on a subset of genes from the same samples. These genes covered several functional clusters such as proteases, structural proteins, inflammatory molecules, and heat shock response. RT-qPCR confirmed significant upregulation (P < 0.05) in all assessed genes: ADAM19, MMP9, COL17A1, LAMA3, VIM, ICAM-1, CSF2, CSF3, IL23a, IL-32, HSPA5, and HSP90B1 (**Figure 2**).

### Functional Enrichment

Gene ontology assessment for biologic processes demonstrated significant enrichment of extracellular structures, epidermal development, and inflammasome activation (response to lipopolysaccharide), as well as various aspects of DNA replication. Functional enrichment for cellular components demonstrated enhancement in notable pathways including extracellular matrix genes and basement membranes genes, while enrichment for molecular function demonstrated notable effects on glycosaminoglycan binding, cytokine binding, integrin binding, and transmembrane receptor protein kinase activity pathways **(** **Supplementary Figure 1** **)**.

### Treatment of Primary Human Keratinocytes With IgG From Laminin-332 Pemphigoid Specific to Each Subunit

Considering the pro-inflammatory and proteolytic effect of IgG autoantibodies from patients with laminin-332 pemphigoid on keratinocytes identified in our initial experiment, we next looked to validate several of the key findings in a larger cohort of patients with laminin-332 pemphigoid. Additionally, we questioned whether the reactivity against specific laminin subunits affects the inflammatory and proteolytic keratinocyte response. We treated PHK with IgG from a separate cohort of patients with reactivity against only either laminin α3 (n=5), laminin β3 (n=4), or laminin γ2 (n=5). Patient demographics are shown in **Table 1**.

**Figure 3:**
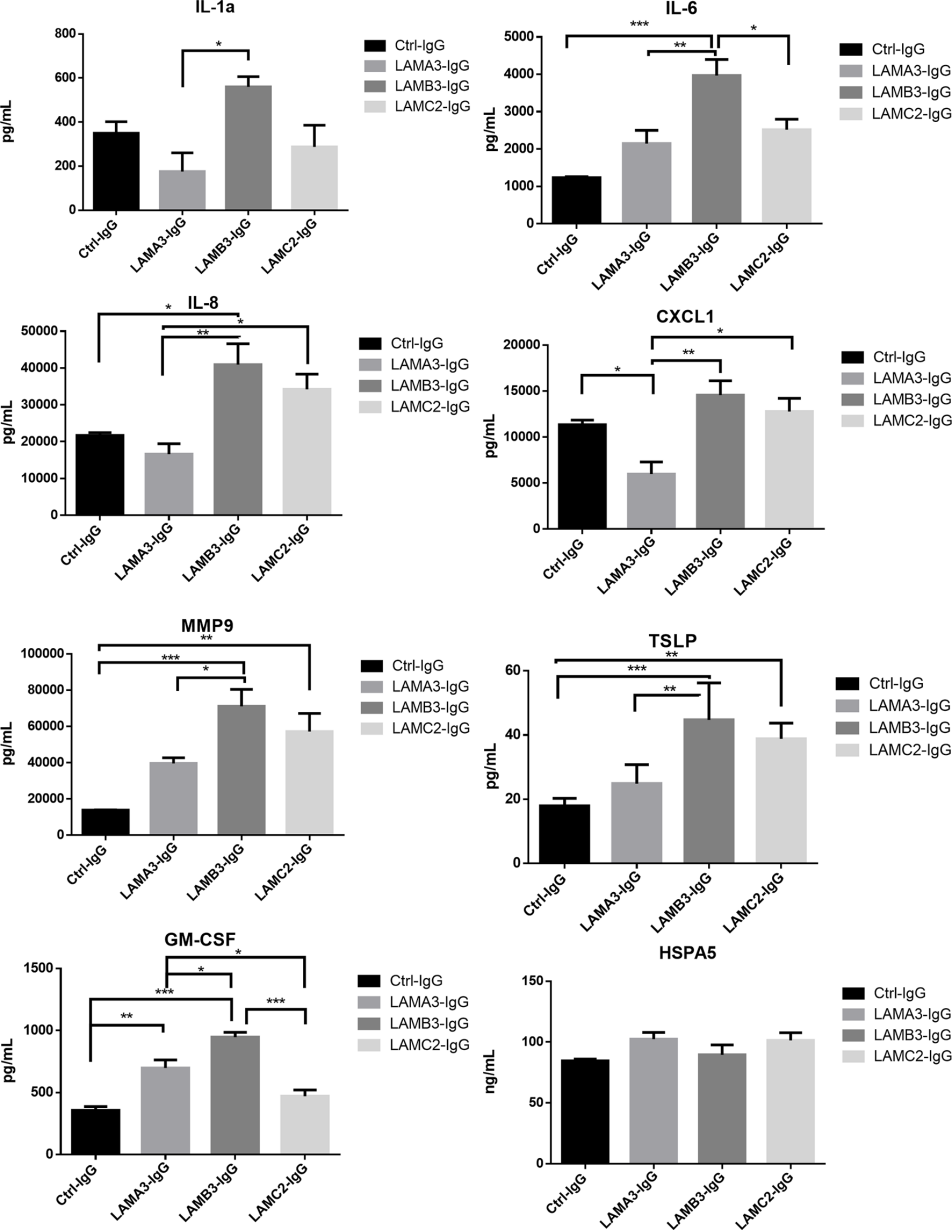
Primary human keratinocytes were treated for 24 hours with 3.5μg/μL of IgG from patients with laminin-332 pemphigoid with autoreactivity against either laminin α3 (n=4-5), laminin β3 (n=4), laminin γ2 (n=4-5), or control (n=4). Luminex and ELISA assays were performed to quantify supernatant protein expression. Bar charts demonstrate significant upregulation of IL-1a, IL-6, IL-8, CXCL1, MMP9, TSLP, and GM-CSF by one way ANOVA (P < 0.05). Bars indicate Tukey’s test for multiple comparisons between groups (*P <0.05, **P < 0.01, ***P < 0.001). HSPA5 levels did not significantly differ. Values shown are for a single experiment.

**Figure 4:**
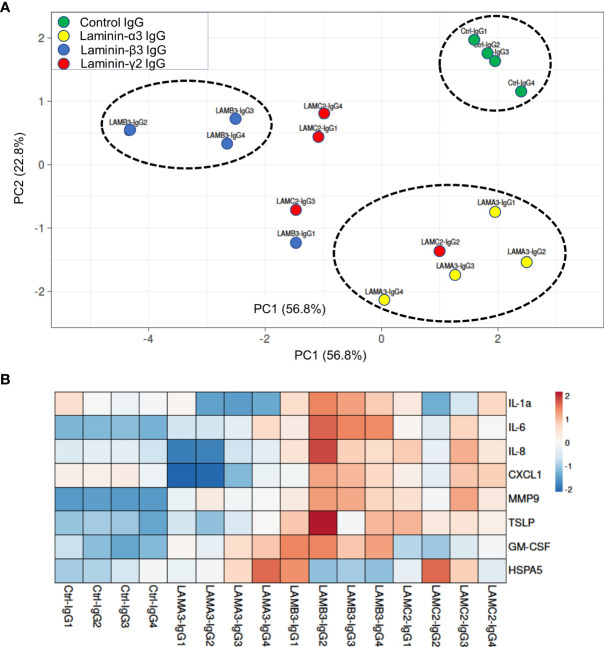
**(A)** Principal component analysis of supernatant protein concentrations spatially represents control IgG versus each laminin subunit, with greatest distinction between laminin β3 autoreactivity and control IgG. Only samples with protein levels for all markers were included in PCA. **(B)** Normalized concentrations of supernatant proteins presented as a heat map demonstrate upregulation of inflammatory markers in laminin β3-IgG treated keratinocytes followed by laminin γ2-IgG treated keratinocytes.

We next evaluated IL-1α, IL-6, IL-8, CXCL1, MMP9, GM-CSF protein expression using Luminex and ELISA assays, confirming overall upregulation. We additionally evaluated TSLP as a pro-inflammatory marker which has been described in BP; this too was elevated at the protein level, though not significantly increased at the mRNA level by RNA-seq. Inflammatory markers were consistently most elevated in patients with reactivity against the laminin β3 subunit (**Figure 3**). We did not find a difference in HSPA5 expression among different groups. Protein expression heat map demonstrates heterogeneity within responses from each subunit, however, heatmap and principal component analysis demonstrate striking distinction between laminin β3 reactivity, and both control and laminin α3 (**Figure 4**). Given the limited sample size and records, we were unable to identify significant association between pro-inflammatory effect of patients’ IgG with their histologic or clinical presentations.

## Discussion

Local inflammatory responses in laminin-332 pemphigoid vary significantly in human disease and in different in vivo models (13–15, 28). Though complement activation can account for a portion of the inflammatory response and is required in some animal models (14), this is at odds with the finding of a predominance of non-complement fixing IgG autoantibodies in patients with laminin-332 pemphigoid (16). The aim of this study was to test the hypothesis that IgG autoantibodies to laminin-332 trigger a pro-inflammatory response in keratinocytes. While in BP, this phenomenon has been well described and accounts for part of the complement-independent mechanism of blistering and inflammatory response (29, 30), it has not previously been described in laminin-332 pemphigoid. We herein demonstrate upregulation at both the mRNA and protein levels of numerous pro-inflammatory cytokines and chemokines in keratinocytes treated with anti-laminin-332 IgG. We additionally demonstrate significant transcriptional upregulation of heat shock proteins and metalloproteases, which have been described to have a contributory mechanism in other autoimmune blistering disorders (31–34). We have identified IgG autoantibodies against the laminin β3 subunit as having the most pro-inflammatory effect, followed by anti-γ2, and anti-α3 laminin. Interestingly, anti-laminin β3 autoantibodies are the least prevalent autoantibodies in laminin-332 pemphigoid (6, 7).

A few limitations must be noted. We selected patients with IgG autoantibodies to each subunit specifically. However, patients often harbor IgG autoantibodies to several domains of laminin-332 concurrently. As such, synergistic effects of autoantibodies targeting different subunits were not identified in this approach. Additionally, the presence of concomitant autoantibodies may contribute a small degree to these findings. For example, BP180 antibodies are known to induce a pro-inflammatory response. As these antibodies were detected by ELISA, but no epidermal staining was appreciated on IIF, two samples were included.

We did not exclude patients who had positive ELISA for BP180/BP230 when looking at laminin subtype. It should be noted that IIF was only positive on dermal side for all of these, so BP180/BP230 autoantibodies are likely at a far lower concentration relative to laminin sub-unit specific antibodies or are not against pathogenic epitopes. Patient LAMA3-IgG3 had antibodies to BP180/BP230 but did not appear to significantly differ from other LAMA3 sera on PCA analysis. When evaluating the protein expression heatmap, the presence of BP180 antibodies do not appear to affect the inflammatory response. In fact, patient LAMB3-IgG1, and LAMA3-IgG3 do not demonstrate particularly high expression relative to the other LAMB3. Thus, the presence of these antibodies appears insufficient to explain our pro-inflammatory findings. While patient LAMC2-IgG3 additionally had autoantibodies against the p200 antigen detected by immunoblot, the patient’s clinical presentation was consistent with laminin-332 mucous membrane pemphigoid. Nevertheless, we cannot rule out a contributory role of p200 antibodies. Analysis of LAMC2 treated patient does not reveal comparable clustering to other subunits. Yet, the most notable ‘outlier’ is patient LAMC2-IgG2, who only expressed autoantibodies to LAMC2. Lastly, we were unable to correlate histopathological findings with keratinocyte cytokine expression levels due to a limited number of specimens with histology.

In addition to identifying potential complement-independent inflammatory mechanisms in laminin-332 pemphigoid, these findings shed light on laminin-332 as an active regulator of cell signalling and function. Extracellular matrix proteins not only serve as structural proteins but affect cell signalling through protein-protein interactions (35). Blockade of laminin α3 and integrin α3β1 interactions are known to lead to keratinocyte differentiation (36). We have recently identified this keratinocyte differentiation to sufficiently induce blistering in 3D skin equivalents, through a protein kinase C and NOTCH dependent pathway (Bao et al., in submission). Notably, RNA-seq in our samples of laminin-332 IgG treated keratinocytes failed to demonstrate keratinocyte differentiation, but rather showed restorative upregulation of hemidesmosomal genes. Whether patient IgG autoantibodies failed to block laminin α3 and integrin α3β1 interaction seen in studies using monoclonal antibodies, or whether IgG autoantibodies to multiple differing epitopes prevent this keratinocyte differentiation requires further study. This does, however, point the importance of polyclonality in patient samples which may be missed when using a monoclonal antibody. We additionally identified distinct inflammatory responses to antibodies against the laminin β3 and γ2 subunit that were absent in patients with laminin α3 autoantibodies. This suggests these subunits may have discrete signalling partners in the BMZ homeostasis from laminin α3.

While we were unable to confirm upregulation of heat shock proteins at the protein level, transcriptional upregulation of HSP90B1, HSP90B2P, HSP90B3P, and HSPA5 was notable. Heat shock protein activation in pemphigoids is well described, and heat shock proteins can be overexpressed in keratinocytes, fibroblasts, or leukocytes. Activation of HSP90, cHSP70, and HSP27 has been described in the stroma of ocular pemphigoid. HSP47 is activated in fibroblasts from MMP and ocular cicatricial pemphigoid (37, 38). However, studies in BP have noted strong Hsp90 overexpression in the epidermis. Similarly, stimulation of keratinocytes with BP-IgG can induce HSP90 expression (39). Inhibition of keratinocyte Hsp90 led to a decrease in BP-IgG induced IL-6 and IL-8 expression (40). Inhibition of Hsp90 has also demonstrated therapeutic efficacy in *in vivo* models of epidermolysis bullosa acquisita, though its mechanism of action appears to be tied towards neutrophil function (41, 42). Thus, the discovery of significant upregulation of Hsp90 genes HSP90B1, HSP90B2P, HSP90B3P in laminin-332 pemphigoid IgG treated may present a possible therapeutic approach.

The role of laminin-332 in carcinogenesis warrants further discussion. Laminin-332 pemphigoid is thought to arise more commonly in patients with underlying malignancy (43). Additionally, laminin-332 is known to play a significant role in tumorigenesis (5, 44–46). Significant research has implicated laminin-332 and integrin signalling in cell migration and invasion in cancer (46). Our study demonstrated direct induction of several inflammatory cytokines by keratinocytes treated with laminin-332 autoantibodies reactive against the β3 and γ2 subunits. While laminin-332 can regulate numerous aspects of tumorigenesis through binding with other BMZ components (47), its ability to regulate local inflammation remains unclear in cancer and is primarily described in inflammatory bowel disease (48–50).

In conclusion, we have characterized the keratinocyte transcriptome as a response to laminin-332 pemphigoid IgG. We for the first time have demonstrated a pro-inflammatory response, similar to that described in keratinocytes treated with IgG autoantibodies from patients with BP. These insights improve our understanding of laminin-332 pemphigoid pathogenesis and laminin-332 biology.

## Data Availability Statement

The datasets presented in this study can be found in online repositories. The names of the repository/repositories and accession number(s) can be found below: https://www.ncbi.nlm.nih.gov/geo/, GSE182644.

## Ethics Statement

The studies involving human participants were reviewed and approved by the ethical committee at the University of Illinois at Chicago, Rush University Medical Center, Philipps University, and Kurume University School of Medicine. The patients/participants provided their written informed consent to participate in this study.

## Author Contributions

KA and LB designed the research study. LB, FS, JL, DD, and PP performed the experiments. LB, XL, HQ, and KA analyzed the data. KA and PP drafted the manuscript. LB, JL, FS, DD, XL, HQ, NI, TH, and MH provided critical revisions. All authors contributed to the article and approved the submitted version.

## Funding

The work was supported in part by the Deutsche Forschungsgemeinschaft (DFG) FOR 2497/TP02 (FS, DD, MH) and the Albert H. and Mary Jane Slepyan Endowed Fellowship (LB).

## Conflict of Interest

The authors declare that the research was conducted in the absence of any commercial or financial relationships that could be construed as a potential conflict of interest.

## Publisher’s Note

All claims expressed in this article are solely those of the authors and do not necessarily represent those of their affiliated organizations, or those of the publisher, the editors and the reviewers. Any product that may be evaluated in this article, or claim that may be made by its manufacturer, is not guaranteed or endorsed by the publisher.

## References

[1] Egan CA Lazarova Z Darling TN Yee C Yancey KB. Anti-Epiligrin Cicatricial Pemphigoid: Clinical Findings, Immunopathogenesis, and Significant Associations. Med (Baltimore) (2003) 82:177–86. doi: 10.1097/01.md.0000076003.64510.00 12792304

[2] Jedlickova H Racovska J Niedermeier A Feit J Hertl M. Anti-Basement Membrane Zone Antibodies in Elderly Patients With Pruritic Disorders and Diabetes Mellitus. Eur J Dermatol (2008) 18:534–8. doi: 10.1684/ejd.2008.0483 18693156

[3] Terra JB Pas HH Hertl M Dikkers FG Kamminga N Jonkman MF. Immunofluorescence Serration Pattern Analysis as a Diagnostic Criterion in Antilaminin-332 Mucous Membrane Pemphigoid: Immunopathological Findings and Clinical Experience in 10 Dutch Patients. Br J Dermatol (2011) 165:815–22. doi: 10.1111/j.1365-2133.2011.10474.x 21692774

[4] Timpl R Tisi D Talts JF Andac Z Sasaki T Hohenester E. Structure and Function of Laminin LG Modules. Matrix Biol (2000) 19:309–17. doi: 10.1016/S0945-053X(00)00072-X 10963991

[5] Marinkovich MP. Tumour Microenvironment: Laminin 332 in Squamous-Cell Carcinoma. Nat Rev Cancer (2007) 7:370–80. doi: 10.1038/nrc2089 17457303

[6] Amber KT Bloom R Hertl M. A Systematic Review With Pooled Analysis of Clinical Presentation and Immunodiagnostic Testing in Mucous Membrane Pemphigoid: Association of Anti-Laminin-332 Igg With Oropharyngeal Involvement and the Usefulness of ELISA. J Eur Acad Dermatol Venereol (2016) 30:72–7. doi: 10.1111/jdv.13397 26446477

[7] Li X Qian H Natsuaki Y Koga H Kawakami T Tateishi C. Clinical and Immunological Findings in 55 Patients With Anti-Laminin 332-Type Mucous Membrane Pemphigoid. Br J Dermatol (2021) 185(2):449–51. doi: 10.1111/bjd.20099 33811327

[8] Lazarova Z Hsu R Yee C Yancey KB. Antiepiligrin Cicatricial Pemphigoid Represents an Autoimmune Response to Subunits Present in Laminin 5 (Alpha3beta3gamma2). Br J Dermatol (1998) 139:791–7. doi: 10.1046/j.1365-2133.1998.02502.x 9892943

[9] Chiorean R Danescu S Virtic O Mustafa MB Baican A Lischka A. Molecular Diagnosis of Anti-Laminin 332 (Epiligrin) Mucous Membrane Pemphigoid. Orphanet J Rare Dis (2018) 13:111. doi: 10.1186/s13023-018-0855-x 29980216 PMC6035451

[10] Amber KT Murrell DF Schmidt E Joly P Borradori L. Autoimmune Subepidermal Bullous Diseases of the Skin and Mucosae: Clinical Features, Diagnosis, and Management. Clin Rev Allergy Immunol (2018) 54:26–51. doi: 10.1007/s12016-017-8633-4 28779299

[11] Bekou V Thoma-Uszynski S Wendler O Uter W Schwietzke S Hunziker T. Detection of Laminin 5-Specific Auto-Antibodies in Mucous Membrane and Bullous Pemphigoid Sera by ELISA. J Invest Dermatol (2005) 124:732–40. doi: 10.1111/j.0022-202X.2005.23646.x 15816831

[12] Lazarova Z Yee C Darling T Briggaman RA Yancey KB. Passive Transfer of Anti-Laminin 5 Antibodies Induces Subepidermal Blisters in Neonatal Mice. J Clin Invest (1996) 98:1509–18. doi: 10.1172/JCI118942 PMC507581 8833897

[13] Lazarova Z Hsu R Yee C Yancey KB. Human Anti-Laminin 5 Autoantibodies Induce Subepidermal Blisters in an Experimental Human Skin Graft Model. J Invest Dermatol (2000) 114:178–84. doi: 10.1046/j.1523-1747.2000.00829.x 10620135

[14] Heppe EN Tofern S Schulze FS Ishiko A Shimizu A Sina C. Experimental Laminin 332 Mucous Membrane Pemphigoid Critically Involves C5ar1 and Reflects Clinical and Immunopathological Characteristics of the Human Disease. J Invest Dermatol (2017) 137:1709–18. doi: 10.1016/j.jid.2017.03.037 28456612

[15] Rose C Schmidt E Kerstan A Thoma-Uszynski S Wesselmann U Käsbohrer U. Histopathology of Anti-Laminin 5 Mucous Membrane Pemphigoid. J Am Acad Dermatol (2009) 61:433–40. doi: 10.1016/j.jaad.2009.02.012 19700013

[16] Hsu R Lazarova Z Yee C Yancey KB. Noncomplement Fixing, Igg4 Autoantibodies Predominate in Patients With Anti-Epiligrin Cicatricial Pemphigoid. J Invest Dermatol (1997) 109:557–61. doi: 10.1111/1523-1747.ep12337073 9326390

[17] Hiroyasu S Ozawa T Kobayashi H Ishii M Aoyama Y Kitajima Y. Bullous Pemphigoid Igg Induces BP180 Internalization *via* a Macropinocytic Pathway. Am J Pathol (2013) 182:828–40. doi: 10.1016/j.ajpath.2012.11.029 PMC3590760 23337823

[18] Zhang Y Hwang BJ Liu Z Li N Lough K Williams SE. BP180 Dysfunction Triggers Spontaneous Skin Inflammation in Mice. Proc Natl Acad Sci USA (2018) 115:6434–9. doi: 10.1073/pnas.1721805115 PMC6016813 29866844

[19] Iwata H Ujiie H. Complement-Independent Blistering Mechanisms in Bullous Pemphigoid. Exp Dermatol (2017) 26:1235–9. doi: 10.1111/exd.13367 28418613

[20] Lazarova Z Salato VK Lanschuetzer CM Janson M Fairley JA Yancey KB. Igg Anti-Laminin-332 Autoantibodies are Present in a Subset of Patients With Mucous Membrane, But Not Bullous, Pemphigoid. J Am Acad Dermatol (2008) 58:951–8. doi: 10.1016/j.jaad.2008.02.035 PMC2517626 18396347

[21] Bao L Tessier C Prigent-Tessier A Li F Buzzio OL Callegari EA. Decidual Prolactin Silences the Expression of Genes Detrimental to Pregnancy. Endocrinology (2007) 148:2326–34. doi: 10.1210/en.2006-1643 17255200

[22] Bao L Li J Perez White BE Patel PM Amber KT. Inhibition of Dipeptidyl-Peptidase 4 Induces Upregulation of the Late Cornified Envelope Cluster in Keratinocytes. Arch Dermatol Res (2021). doi: 10.1007/s00403-021-02249-4 PMC9285643 34089377

[23] Kim D Paggi JM Park C Bennett C Salzberg SL. Graph-Based Genome Alignment and Genotyping With HISAT2 and HISAT-Genotype. Nat Biotechnol (2019) 37:907–15. doi: 10.1038/s41587-019-0201-4 PMC7605509 31375807

[24] Trapnell C Roberts A Goff L Pertea G Kim D Kelley DR. Differential Gene and Transcript Expression Analysis of RNA-Seq Experiments With Tophat and Cufflinks. Nat Protoc (2012) 7:562–78. doi: 10.1038/nprot.2012.016 PMC3334321 22383036

[25] Anders S Huber W. Differential Expression Analysis for Sequence Count Data. Genome Biol (2010) 11:R106. doi: 10.1186/gb-2010-11-10-r106 20979621 PMC3218662

[26] Alexa A Rahnenführer J Lengauer T. Improved Scoring of Functional Groups From Gene Expression Data by Decorrelating GO Graph Structure. Bioinformatics (2006) 22:1600–7. doi: 10.1093/bioinformatics/btl140 16606683

[27] Metsalu T Vilo J. Clustvis: A Web Tool for Visualizing Clustering of Multivariate Data Using Principal Component Analysis and Heatmap. Nucleic Acids Res (2015) 43:W566–70. doi: 10.1093/nar/gkv468 PMC4489295 25969447

[28] Babicki S Arndt D Marcu A Liang Y Grant JR Maciejewski A. Heatmapper: Web-Enabled Heat Mapping for All. Nucleic Acids Res (2016) 44:W147–53. doi: 10.1093/nar/gkw419 PMC4987948 27190236

[29] Schmidt E Reimer S Kruse N Jainta S Bröcker EB Marinkovich MP. Autoantibodies to BP180 Associated With Bullous Pemphigoid Release Interleukin-6 and Interleukin-8 From Cultured Human Keratinocytes. J Invest Dermatol (2000) 115:842–8. doi: 10.1046/j.1523-1747.2000.00141.x 11069622

[30] Natsuga K Nishie W Shinkuma S Ujiie H Nishimura M Sawamura D. Antibodies to Pathogenic Epitopes on Type XVII Collagen Cause Skin Fragility in a Complement-Dependent and -Independent Manner. J Immunol (2012) 188:5792–9. doi: 10.4049/jimmunol.1003402 22523387

[31] Holstein J Solimani F Baum C Meier K Pollmann R Didona D. Immunophenotyping in Pemphigus Reveals a T(H)17/T(FH)17 Cell-Dominated Immune Response Promoting Desmoglein1/3-Specific Autoantibody Production. J Allergy Clin Immunol (2021) 147:2358–69. doi: 10.1016/j.jaci.2020.11.008 33221382

[32] Tukaj S Zillikens D Kasperkiewicz M. Heat Shock Protein 90: A Pathophysiological Factor and Novel Treatment Target in Autoimmune Bullous Skin Diseases. Exp Dermatol (2015) 24:567–71. doi: 10.1111/exd.12760 25980533

[33] Nishie W. Collagen XVII Processing and Blistering Skin Diseases. Acta Derm Venereol (2020) 100:adv00054. doi: 10.2340/00015555-3399 32039455 PMC9128997

[34] Akiyama M Hiroyasu S Turner CT Richardson KC Granville DJ. Proteases in Pemphigoid Diseases. Br J Dermatol (2019) 10:1454. doi: 10.3389/fimmu.2019.01454 PMC6607946 31297118

[35] Park EJ Myint PK Ito A Appiah MG Darkwah S Kawamoto E. Integrin-Ligand Interactions in Inflammation, Cancer, and Metabolic Disease: Insights Into the Multifaceted Roles of an Emerging Ligand Irisin. Front Cell Dev Biol (2020) 8:588066. doi: 10.3389/fcell.2020.588066 33195249 PMC7649757

[36] Tayem R Niemann C Pesch M Morgner J Niessen CM Wickström SA. Laminin 332 Is Indispensable for Homeostatic Epidermal Differentiation Programs. J Invest Dermatol (2021) 141(11):2602–10.e3. doi: 10.1016/j.jid.2021.04.008 33965403

[37] Razzaque MS Ahmed AR. Collagens, Collagen-Binding Heat Shock Protein 47 and Transforming Growth Factor-Beta 1 Are Induced in Cicatricial Pemphigoid: Possible Role(s) in Dermal Fibrosis. Cytokine (2002) 17:311–6. doi: 10.1006/cyto.2002.1020 12061838

[38] Razzaque MS Foster CS Ahmed AR. Role of Collagen-Binding Heat Shock Protein 47 and Transforming Growth Factor-Beta1 in Conjunctival Scarring in Ocular Cicatricial Pemphigoid. Invest Ophthalmol Vis Sci (2003) 44:1616–21. doi: 10.1167/iovs.02-0644 12657600

[39] Tukaj S Kleszczyński K Vafia K Groth S Meyersburg D Trzonkowski P. Aberrant Expression and Secretion of Heat Shock Protein 90 in Patients With Bullous Pemphigoid. PLoS One (2013) 8:e70496. doi: 10.1371/journal.pone.0070496 23936217 PMC3728143

[40] Tukaj S Grüner D Zillikens D Kasperkiewicz M. Hsp90 Blockade Modulates Bullous Pemphigoid Igg-Induced IL-8 Production by Keratinocytes. Cell Stress Chaperones (2014) 19:887–94. doi: 10.1007/s12192-014-0513-8 PMC4389849 24796797

[41] Kasperkiewicz M Müller R Manz R Magens M Hammers CM Somlai C. Heat-Shock Protein 90 Inhibition in Autoimmunity to Type VII Collagen: Evidence That Nonmalignant Plasma Cells Are Not Therapeutic Targets. Blood (2011) 117:6135–42. doi: 10.1182/blood-2010-10-314609 21490339

[42] Tukaj S Hellberg L Ueck C Hänsel M Samavedam U Zillikens D. Heat Shock Protein 90 is Required for Ex Vivo Neutrophil-Driven Autoantibody-Induced Tissue Damage in Experimental Epidermolysis Bullosa Acquisita. Exp Dermatol (2015) 24:471–3. doi: 10.1111/exd.12680 25739426

[43] Florea F Koch M Hashimoto T Sitaru C. Autoimmunity Against Laminins. Clin Immunol (2016) 170:39–52. doi: 10.1016/j.clim.2016.07.021 27464450

[44] Meireles Da Costa N Mendes FA Pontes B Nasciutti LE Ribeiro Pinto LF Palumbo Júnior A. Potential Therapeutic Significance of Laminin in Head and Neck Squamous Carcinomas. Cancers (Basel) (2021) 13(8):1890. doi: 10.3390/cancers13081890 33920762 PMC8071176

[45] Rousselle P Scoazec JY. Laminin 332 in Cancer: When the Extracellular Matrix Turns Signals From Cell Anchorage to Cell Movement. Semin Cancer Biol (2020) 62:149–65. doi: 10.1016/j.semcancer.2019.09.026 31639412

[46] Tsuruta D Kobayashi H Imanishi H Sugawara K Ishii M Jones JC. Laminin-332-Integrin Interaction: A Target for Cancer Therapy? Curr Med Chem (2008) 15:1968–75. doi: 10.2174/092986708785132834 PMC2992754 18691052

[47] Baba Y Iyama KI Hirashima K Nagai Y Yoshida N Hayashi N. Laminin-332 Promotes the Invasion of Oesophageal Squamous Cell Carcinoma *via* PI3K Activation. Br J Cancer (2008) 98:974–80. doi: 10.1038/sj.bjc.6604252 PMC2266844 18283320

[48] Spenlé C Hussenet T Lacroute J Lefebvre O Kedinger M Orend G. Dysregulation of Laminins in Intestinal Inflammation. Pathol Biol (Paris) (2012) 60:41–7. doi: 10.1016/j.patbio.2011.10.005 22100883

[49] Condorelli AG Dellambra E Logli E Zambruno G Castiglia D. Epidermolysis Bullosa-Associated Squamous Cell Carcinoma: From Pathogenesis to Therapeutic Perspectives. Int J Mol Sci (2019) 20(22):5707. doi: 10.3390/ijms20225707 PMC6888002 31739489

[50] Neagu M Constantin C Caruntu C Dumitru C Surcel M Zurac S. Inflammation: A Key Process in Skin Tumorigenesis. Oncol Lett (2019) 17:4068–84. doi: 10.3892/ol.2018.9735 PMC6444305 30944600

