# Supplementary material for: Subunit-Specific Reactivity of Autoantibodies Against Laminin-332 Reveals Direct Inflammatory Mechanisms on Keratinocytes
Source: Front Immunol. 2021 Nov 25;12:775412. doi: 10.3389/fimmu.2021.775412 (PMC8655097; doi:10.3389/fimmu.2021.775412)
Supplement: Supplementary file 1 [file DataSheet_1.docx]

Supplementary Material

# Supplementary Tables and Figures

## Supplementary Tables

**Supplementary Table 1.** Human primers used for real-time RT-PCR.

**Gene name Forward primer 5'->3' Reverse primer 5'->3'**

ADAM19 GCC AGG CCT ACT GCT ACA AC GTC TCC TGC CAC ATT CAC CT

COL17A1 TCA GCA CAT CCT CCT CAG TG TCT GGG GCA TGT TTT TCT TC

CSF2 CAG CCA CTA CAA GCA GCA CT AAG GGG ATG ACA AGC AGA AA

CSF3 CTT CGC CTC TGC TTT CCA G GGC AAG GTG GCG TAG AAC

HSPA5 TAG CGT ATG GTG CTG CTG TC TGA CAC CTC CCA CAG TTT CA

HSP90B1 TAG CCA AAT CTG GGA CAA GC AGA AAC CGA CAC CAA ACT GG

ICAM1 TGT CCC CCT CAA AAG TCA TC TAG GCA ACG GGG TCT CTA TG

IL23A TTC TCT GCT CCC TGA TAG CC GAC TGA GGC TTG GAA TCT GC

IL32 CCG AAG GTC CTC TCT GAT GA AGG TGT CCC ACA GTG TCC TC

LAM3 ACT GAG GAT CCC TGT GTG GA CTG GTC AGG ACA ACC ATT CA

MMP9 TCT TCC CTG GAG ACC TGA GA ATT TCG ACT CTC CAC GCA TC

NOTCH1 AGA GAG CTC CTG CTT CAA CG GTC GCA CTC ATT GAC ATC GT

VIM GGC TCA GAT TCA GGA ACA GC TTG GCA GCC ACA CTT TCA TA

**Supplementary Table 2**. Complete list of differentially expressed genes. (Excel file attached).

## Supplementary Figures


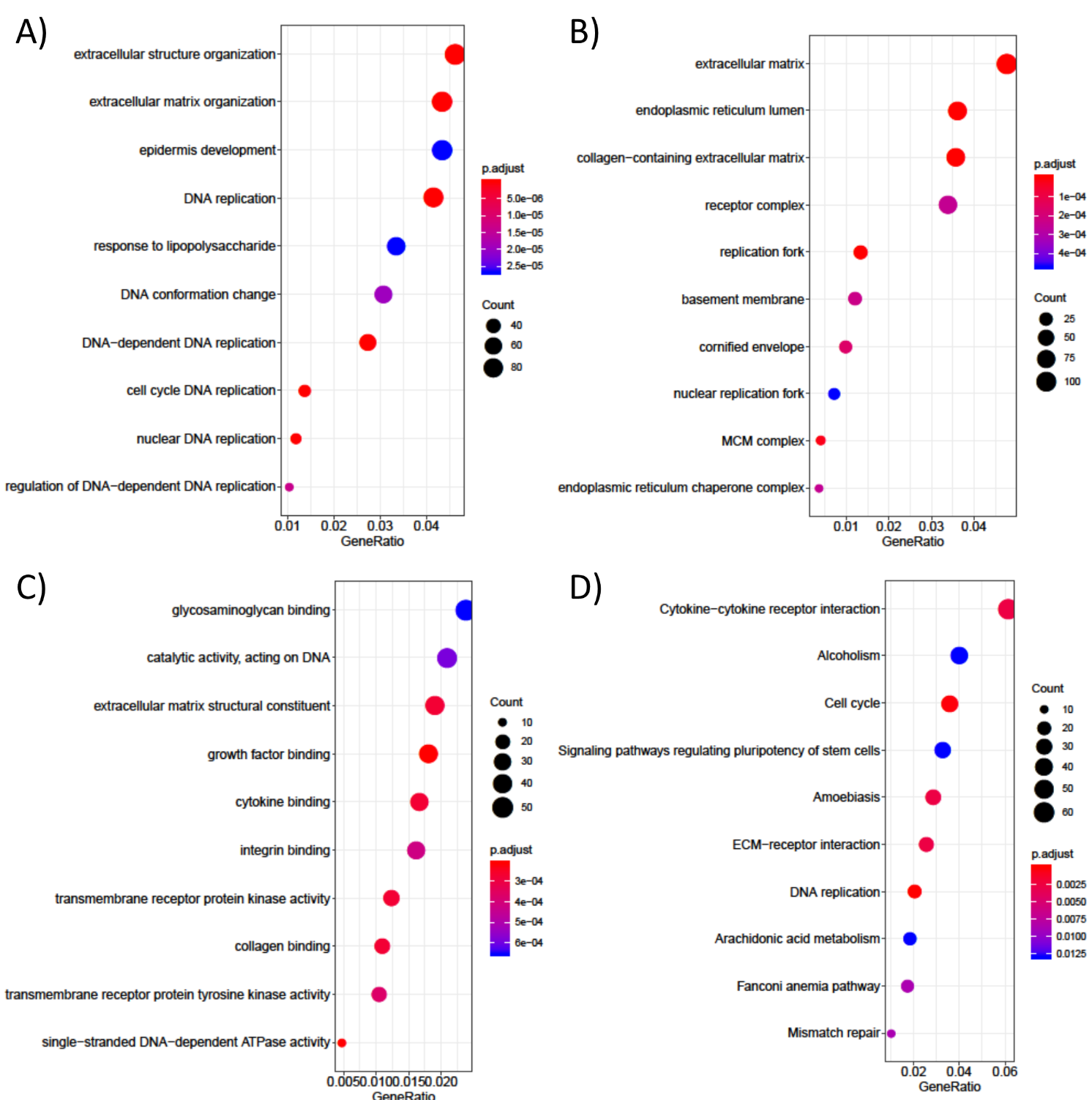


**Supplementary Figure 1.** Gene enrichment of laminin-332 IgG versus control-IgG treated primary keratinocytes demonstrating gene ontology for A) Biological process, B) cellular component, C) Molecular function, as well as D) KEGG enrichment.
